# Cereal–Pea Intercropping Reveals Variability in the Relationships among Yield, Quality Parameters, and Obligate Pathogens Infection in Wheat, Rye, Oat, and Triticale, in a Temperate Environment

**DOI:** 10.3390/plants12112067

**Published:** 2023-05-23

**Authors:** Radivoje Jevtić, Vesna Župunski, Milosav Grčak, Dragan Živančev, Desimir Knežević

**Affiliations:** 1 Institute of Field and Vegetable Crops, Maksima Gorkog 30, 21000 Novi Sad, Serbia; 2 Faculty of Agriculture, University of Priština—Kosovska Mitrovica, Kopaonička bb, 38219 Lešak, Serbia

**Keywords:** intercropping, yield, TKW, crude protein, cereals, pea

## Abstract

Widespread usage of intercropping systems has been limited because of a lack of knowledge about the key factors that affect the performance of intercrop components. We used general linear modelling to explain the effect of different cropping systems on the relationships among yield, thousand kernel weight (TKW), and crude protein of cereal crops under the same agro-ecological conditions and naturally occurring inocula of obligate pathogens. The results of our study showed that the yield variation under extreme fluctuations in climatic conditions could be lowered through intercropping cultivation. The disease indices of leaf rust and powdery mildew were highly dependent on the type of cultivation. The relationships among the levels of pathogenic infection and yield performances were not straightforward and were highly dependent on the yielding potentials of the cultivars. Our study indicated that changes in yield, TKW, and crude protein, as well as their relationships during intercropping cultivation, were cultivar specific and, therefore, not the same among all cereal crops exposed to the same agro-ecological conditions.

## 1. Introduction

Conventional agriculture of sole (i.e., monocultured) crops provides high yields, but it also leads to environmental contamination, soil erosion, and disease resistance to pesticides [1,2]. In addition, extreme fluctuations in climatic conditions have been associated with CO_2_ emissions as a result of the usage and the production of nitrogen fertilizers [3,4]. It has been estimated that 13.4% of the total greenhouse gasses emitted by the agricultural industry have been caused by the production and use of nitrogen fertilizers [5].

The interest in the cultivation of cereal–legume intercrops has increased alongside the increasing demand for the development of agro-ecosystems that can combine high crop productivity with reduced input levels [6,7]. Integrated arable farming systems (IAFS) were introduced as alternatives to intensive farming systems, and they were based on concepts of integrated production (IP) [4,8,9]. The conceptual framework of IP was published for the first time in 1993 by the IOBC Commission on IP Guidelines and Endorsements and was defined as follows: a “farming system that produces high-quality food and other products by using natural resources and regulating mechanisms to replace polluting inputs and to secure sustainable farming” [10]. The diversification of agro-ecosystems by increasing the number of grown species was proposed as a solution for the preservation of agro-ecosystems [11,12,13,14].

Intercropping of legumes and cereals was reported as a promising tool for increasing land use through greater light capture and complementary nutrient acquisition [15,16,17,18]. The complementarity of the usage of N sources by components of the intercrops was considered to be of great importance, especially where mineral N was a limited resource [2]. In addition, the beneficial effects of intercropping on weed control, lodging resistance, yield stability, grain protein concentration, and pest-and-disease management were reported in a comparison with sole crop cultivation [2,11,19,20,21,22,23,24,25,26,27]. The control of the most important obligate pathogens in cereal crops, such as the causal agents of powdery mildew and rust, has been a great concern in cereal crop production. The yield losses of the winter wheat caused by powdery mildew were predicted to reach up to 45% [28], while rust diseases could cause a yield reduction up to 60% or more among the genetic collections used as the phenotype platforms for disease resistance/susceptibility testing [29].

Timaeus et al. [4] pointed out that studies on the multifunctional perspective of species mixtures were missing since the majority of investigations had been focused only on certain aspects of cropping system performance. Annicchiarico et al. [30] also reported that the mutual interaction between the species resulted in an unstable dynamic equilibrium in intercropping systems that made it significantly challenging to identify suitable characters for intercropped cultivars, especially if they had been tested in pure stand cultivation. Consequently, the inability of crop management to control the variability in the percentage of each species in the harvested mixture, and the lack of knowledge regarding the individual crops performance in intercropping systems has been a major drawback to the widespread usage of intercropping cultivation [31].

Since the performance of different intercropped cereals with legumes has rarely been investigated under the same agro-ecological conditions and using the same experimental designs, our study aimed to investigate how different cultivation practices, under the same the agro-ecological conditions and with the naturally occurring inocula of obligate pathogens, would affect the relationships among yield, thousand kernel weight (TKW), and crude protein of wheat, rye, triticale, and oat. Consequently, the objectives of this study were to determine the following: (1) if there was a difference in the relationships among obligate pathogens, yield, yield components, and crude protein among different cereal crops that had been cultivated in pure stand and intercropping systems; (2) if cereal crops had different contributions to the land equivalent ratios (LERs) when cultivated under the same agro-ecological conditions; and (3) if nitrogen usage would differ among cereal crops in cereal–pea intercropping systems.

## 2. Results

Our general linear modelling (GLM) confirmed that cultivation practices significantly affected the yield (*p* < 0.001), TKW (*p* < 0.001), and the crude protein content (*p* < 0.001), as well as the disease index, of leaf rust (*p* < 0.001) and powdery mildew (*p* < 0.001) (Table 1). In addition to the cultivation practice, the GLM also showed that the year and variety significantly affected the yield (*p* = 0.003; *p* < 0.001), TKW (*p* = 0.006; *p* < 0.001), the crude protein (*p* < 0.001; *p* < 0.001), and the disease index of leaf rust (*p* = 0.009; *p* < 0.001) (Table 1). The differences among the effects of the interactions (year × variety, year × cultivation, variety × cultivation, and year × variety × cultivation), on all examined traits, indicated the various responses of cereal crops under different cultivation practices in the two-year-long experiment. The variety also significantly affected the levels of infection of powdery mildew (*p* < 0.001) in cereal crops (Table 1). In this study, the peas were considered as the alternative crops intercropped with cereals. We did not observe the beneficial effects of intercropping on the yield and the crude protein contents in peas (Table S1, Figure S1); therefore, we focused more on the beneficial effects of peas on cereal crop production.

**Figure 1:**
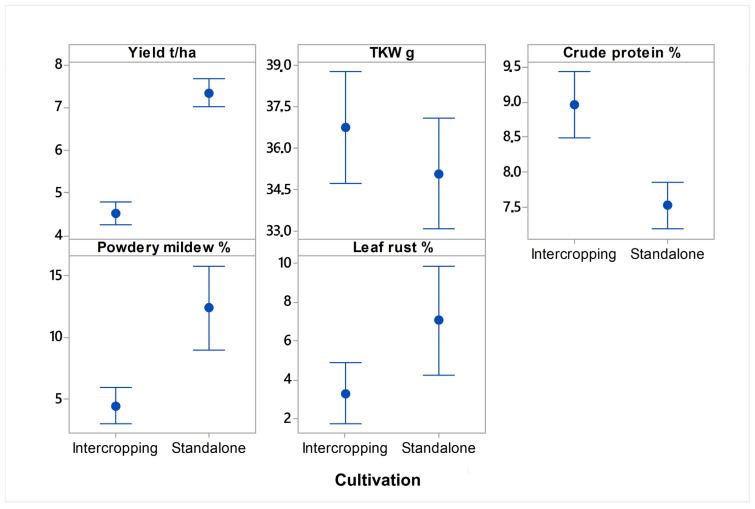
Averages of yield, TKW, crude protein, disease index of leaf rust, and disease index of powdery mildew, in wheat, rye, oat, and triticale cultivated in intercropping and standalone cultivation systems. The interval plot display consists of a mean symbol with a 95% confidence interval bar.

On average, when all the cereal crops were considered, the yields (7.3 t/ha), the DI of leaf rust (7.1%), and the DI of powdery mildew (12.4%) were significantly higher in pure stand cultivation than in intercropping cultivation with pea (Figure 1). In intercropping cultivation, the averages of the yields, the DI of leaf rust, and the DI of powdery mildew over two years equaled 4.5 t/ha, 3.3%, and 4.5%, respectively. Contrary to those results, the TKW (36.8 g) and the crude protein (9%) of the cereal crops were higher in the intercropping systems than the TKW (35.1 g) and the crude protein (7.5%) in pure stand cultivation (Figure 1). More specifically, the averages of the yield, the TKW, the crude protein, the DI of leaf rust, and the DI of powdery mildew, per each crop, year, and cultivation practice, are presented in Table 2. Their associations were analyzed in more detail, as discussed in the following paragraphs.

### 2.1. Difference in Effects of Year and Cultivation Practices on Yield and TKW of Cereal Crops

**Figure 2:**
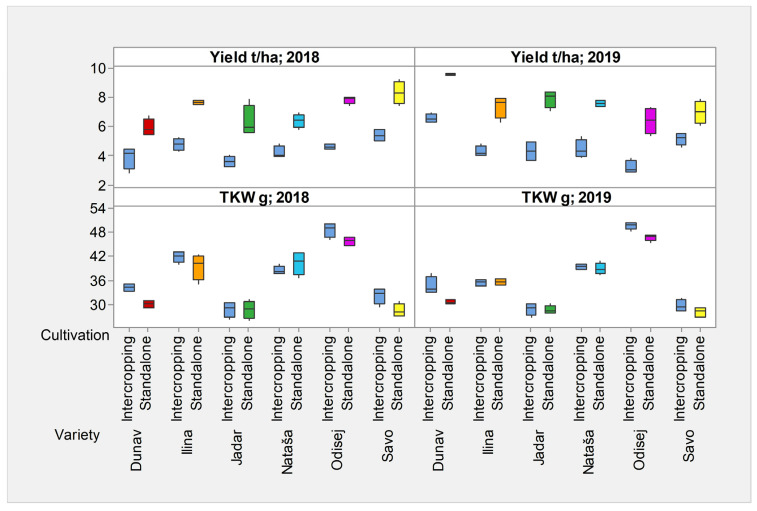
Box plots showing yield and TKW of wheat, rye, oat, and triticale, cultivated in intercropping and standalone cultivation systems in 2018 and 2019. Dunav is spring oat; Ilina is winter wheat; Jadar is winter oat; Nataša is spring wheat; Odisej is winter triticale; and Savo is winter rye.

In general, intercropping decreased the yields of all cereal crops in both years (Figure 2, Table S2). The effects of intercropping cultivation on the TKW results were different from those on yield (Figure 2, Table S2). As opposed to yield, they affected the TKW increment in the winter triticale Odisej and the spring oat Dunav (Table S2). The exceptions were the spring wheat Nataša and the winter oat Jadar, which did not exhibit any advantage in TKW performance in 2018 or 2019 (Table S2). Interestingly, the differences in the TKWs over the two growing seasons were prominent only for the variety Ilina, indicating higher values in 2018 than in 2019, under both types of cultivation (Figure 2). The TKWs of the winter wheat Ilina and winter rye Savo were higher when intercropped than when cultivated alone, but the differences were not significant when both years were considered (Table S2).

Although the year × variety significantly affected both the yield (*p* < 0.001) and the TKW (*p* < 0.001), the year × variety × cultivation practice significantly influenced only yield (*p* = 0.033) (Table 1). Indeed, yield differences of a single variety in 2018 and 2019 were greater than differences in TKW, and cultivation practice affected the extent of these differences in examined cultivars and years (Figure 2).

Although the intercropping decreased the yield of all the studied cereal crops, the extent of the yield decrement was not uniform and was the greatest (>3 t/ha) in winter wheat, Ilina; the winter oat, Jadar; and the winter triticale, Odisej.

### 2.2. Potentials of Cereal–Pea Intercropping Systems for Regulating Powdery Mildew and Leaf Rust Infections

In our study, both growing seasons were favorable for the occurrence of obligate pathogens. The average temperatures in April ranged from 13.4 (2019) to 17.2 °C (2018), which was in accordance with the temperatures reported to be conducive for the germination of leaf rust urediniospores [32]. The total rainfall and relative humidity in April did not differ in the two growing seasons, and the relative humidity higher than 60% provided conditions for leaf rust occurrence in both 2018 and 2019. The climatic conditions during the two-year study were favorable for powdery mildew occurrence, as well. It was reported that the optimal temperatures for the conidia germination of powdery mildew ranged from 1 to 30 °C, without the presence of water, while optimal temperatures for infection ranged from 5 to 30 °C [33]. Although the climatic conditions supported the occurrence of obligate pathogens in both 2018 and 2019, not all cereal crops showed the same levels of susceptibility.

In pure stand cultivation, winter wheat Ilina had the highest levels of susceptibility to powdery mildew, showing DIs of 35% in 2018 and 30% in 2019. The highest susceptibility to leaf rust was found in the winter rye Savo, with DIs of 12.5% in 2018 and 27.5% in 2019 (Table 2). The relationships among powdery mildew and leaf rust in winter triticale Odisej were not the same in the two growing seasons. Leaf rust predominated over powdery mildew in 2018 with a DI of 15%, while in 2019, powdery mildew was predominant with a DI of 15%. The spring wheat Nataša was more infected with leaf rust (DI = 12.5%) than with powdery mildew (DI = 3.75%) in 2019, while in 2018, neither pathogen infected that variety (Table 2).

**Figure 3:**
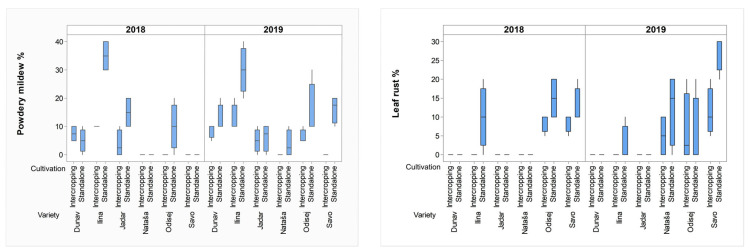
Box plots of disease indices of powdery mildew and leaf rust on wheat, rye, oat, and triticale, in intercropping and standalone cultivation systems in 2018 and 2019. Dunav is spring oat; Ilina is winter wheat; Jadar is winter oat; Nataša is spring wheat; Odisej is winter triticale; and Savo is winter rye.

Cultivation practices significantly affected the risk of infection by leaf rust (*p* < 0.001) and powdery mildew (*p* < 0.001) in the cereal crops (Table 1). Cereal–pea intercropping lowered the disease index of powdery mildew in the winter wheat Ilina to 10% (2018) and 12.5% (2019). The same was true for the winter rye Savo when its leaf rust infection decreased from 27.5% to 11.3% in 2019. (Figure 3, Table S3). Intercropping lowered the DI of obligate pathogens below 10% even when pathogens coexisted on one cultivar (spring wheat Nataša, winter triticale Odisej) (Figure 3).

GLM analysis also showed that the interaction of year × variety × cultivation significantly influenced the disease indices of both leaf rust (*p* = 0.021) and powdery mildew (*p* = 0.001) (Table 1). These results indicated that the disease indices of the obligate pathogens were highly dependent not only on the environmental factors influencing the life cycles of the pathogens and the susceptibility of the cereal crops, but also on the type of cultivation.

Our results also indicated that the relationship between the level of pathogenic infection and yield performance was not straightforward and highly dependent on the yielding potentials of the cultivars. This indicated that investigations into the effects of practices on disease control could not be fully understood without understanding how the impact of different levels of pathogenic infections affect yield losses. In pure stand cultivation in 2018, the winter wheat Ilina was infected with powdery mildew (DI = 35%), but the yield (7.6 t/ha) was higher than the yield of the spring wheat Nataša (6.4 t/ha) that was not infected with obligate pathogens (Table 2). In the same year, the DI of the obligate pathogens identified in intercropping cultivation did not exceed 10% in both varieties, but the yield of Ilina (4.7 t/ha) was also greater than the yield of Nataša (4.2 t/ha) (Table 2). This indicated that factors other than obligate pathogens had resulted in differences in the yields of these two varieties in pure stand cultivation in 2018 (Table S4). 

### 2.3. Effect of Intercropping Systems on Nitrogen Usage of Cereal Crops and Contribution of Cereal Crops to the Land Equivalent Ratio

**Figure 4:**
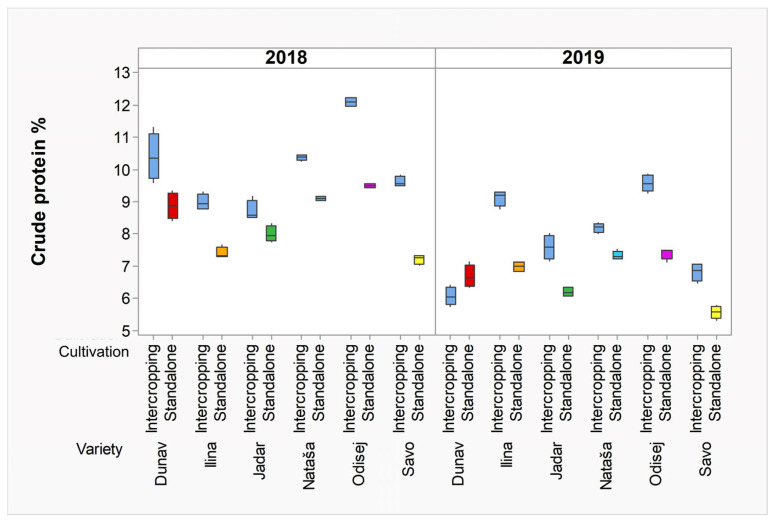
Box plots illustrating crude protein contents of wheat, rye, oat, and triticale, cultivated in intercropping and standalone cultivation systems in 2018 and 2019. Dunav is spring oat; Ilina is winter wheat; Jadar is winter oat; Nataša is spring wheat; Odisej is winter triticale; and Savo is winter rye.

The nitrogen usage of all cereal crops was more prominent in intercropping systems with pea, in both 2018 and 2019, although the overall crude protein contents were less in 2019 than in 2018 (Figure 4). The year, the variety, the cultivation practices, and their interactions (year × variety; year × cultivation; variety × cultivation and year × variety × cultivation) were all shown to be significantly influential factors (*p* < 0.001) (Table 1 and Table S5).

**Figure 5:**
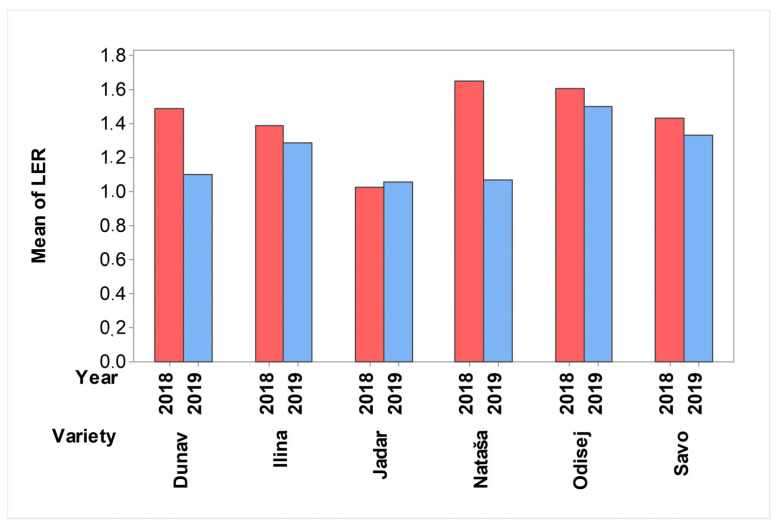
Land equivalent ratio of wheat, rye, oat, and triticale, in 2018 and 2019. Dunav is spring oat; Ilina is winter wheat; Jadar is winter oat; Nataša is spring wheat; Odisej is winter triticale; and Savo is winter rye.

Although all the cereal crops exhibited lower yields when intercropped with pea, the land equivalent ratio exceeded one for all of them, indicating the higher land usage of the intercropping systems. The highest value of the land equivalent ratio of the yields (1.65) was in 2018, when the spring wheat Nataša was intercropped with pea (Figure 5). In the same year, the intercropping of the winter oat Jadar with pea yielded an LER that was only 1.03. The intercropping of the winter oat Jadar with pea reached an LER of 1.06 in 2019. In general, the LER values were lower in 2019 than in 2018, but the contributions of the cereal crops to the LERs were diverse. In 2019, the spring wheat Nataša was associated with a lower LER (1.07) than in 2018, while the intercropping of the winter triticale Odisej with pea resulted in a high LER in both 2018 (1.6) and 2019 (1.5) (Figure 5).

**Figure 6:**
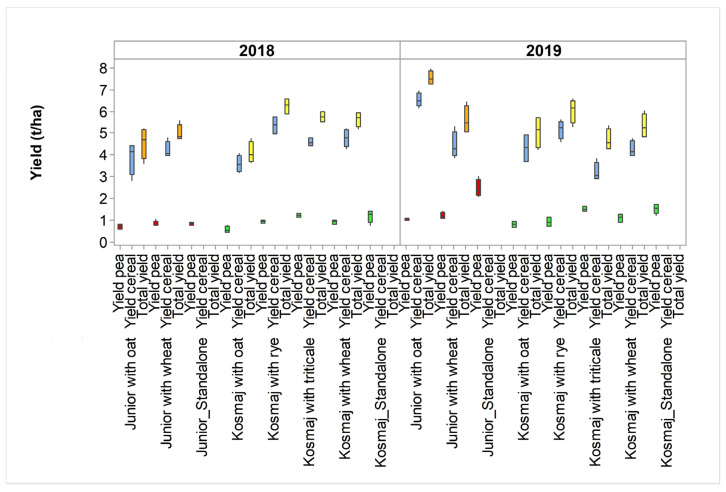
Box plots illustrating total and partial yields of each intercropped species in 2018 and 2019. Junior is spring pea; Kosmaj is winter pea; Dunav is spring oat; Ilina is winter wheat; Jadar is winter oat; Nataša is spring wheat; Odisej is winter triticale; and Savo is winter rye.

We noted that the LER of the yield indicated the sum of the partial LER values for cereal and legume crops (Table S6). It did not necessarily indicate which crop would contribute the most to the absolute values of the total yields (which were expressed as sums of the yields of the intercropped species), nor did it indicate which combinations of intercropped varieties would provide the highest total yields. For example, the contributions of the cereal crops to the total yields were greater than that of the peas (Figure 6), yet the partial LERs of the cereals were usually lower than that of the peas (Table S6).

## 3. Discussion

At present, studies have mainly focused on the effects of different environmental factors on the yield and quality parameters of individual species in intercropping systems, but little has been known about the impact of different cultivation practices under the same agro-ecological conditions on the relationships among yield, TKW, and crude protein, in various cereal crops. Our study revealed key factors affecting yield, TKW, and crude protein parameters and their interactions in wheat, triticale, oat, and rye, which had been cultivated under the same agro-ecological conditions and with different cultivation practices.

### 3.1. The Variable Effects of Year and Cultivation Practices on Yields and TKWs of Cereal Crops

The lower yield performances of cereal crops in intercropping cultivation supported a previous report by Hauggaard-Nielsen et al. [34], who indicated that the grain yield of wheat had linearly decreased with a reduction of wheat density in intercropping systems. In contrast, however, Li et al. [35] noted that the intercropping of wheat with broad beans had comparable yields with pure stand cultivation. However, in Li’s study, the plants/m^2^ of wheat under two types of cultivation were the same. Grain yield is the product of three components: ears/m^2^, grains/ear, and individual grain weight; therefore, in future studies, the effect of the sowing rate on yield achievements in intercropping cultivation should not be analyzed as an individual factor but through its effect on each yield component. In addition, in our study, the effect of intercropping on TKW was different to yield and resulted in a significant increase in the TKWs of the winter triticale Odisej and the spring oat Dunav, as well as a non-significant increase in the winter wheat Ilina and the winter rye Savo.

These results indicated that the intercropping of cereal crops with pea had not affected changes in their yields and TKWs in the same manner. The greater influence of the interactions between the year × variety × cultivation practices on the yield, as compared to the TKWs, raised questions regarding which parameters should be monitored in intercropping systems in order to predict the yield and yield components. Jevtić et al. [36] reported the partial or lack of correlation between the yield and TKW results from partially or non-correlated changes in factors affecting wheat development at different growth stages. It was reported that the factors affecting yield were more likely related to the period before and just after anthesis, while the kernel weight was more likely associated with factors occurring during the grain-filling period [37]. Sugár et al. [38] indicated that TKW could compensate for the crop losses under unfavorable weather conditions, to a certain extent. In the study by Harasim et al. [39], the TKW contributed to the grain yields, from −0.4% to 13.3%, depending on the growing season.

The significant increase in the TKW of the spring oat Dunav, when intercropped with pea, agreed with the results of Neugschwandtner [40], which had indicated that the TKW of oats could increase with decreasing shares of oats in oat–pea intercropping systems. Since it was not the objective of our study to determine the mechanism of resource usage in cereal crops (N accumulation in the rhizosphere of pea, transferring of symbiotically fixed N_2_ to cereal crops, or complementary usage of inorganic N in the soil), we could not determine whether N availability had affected the increased TKWs in certain cultivars or whether this was related to some other mechanism. In previous studies, the association between inorganic N content in the soil and the TKW was either negatively correlated or not correlated at all [38,41]. Sugár et al. [38] reported that N fertilization had affected the negative association between yield and TKW by increasing the yield and decreasing the TKW. In the same study by Sugár et al. [38], the correlation between TKW and yield was not deemed significant in the studied trials without N fertilization. In the study by Protić et al. [41], rising levels of N input also decreased the TKW results. However, Xu et al. [42] reported that TKW had been affected by the cropping system but not by N management. Consequently, we speculated in our study that intercropping cultivation had increased the TKW of the winter triticale Odisej and the spring oat Dunav indirectly through mechanisms affecting yield and/or other yield components.

The higher temperatures at the time of flowering in 2018 resulted in a decreased yield of the spring oat Dunav, the spring wheat Nataša, and the winter oat Jadar, in both types of cultivation. In addition, the yields of the winter triticale Odisej, the winter wheat Ilina, and the winter rye Savo were higher in 2018 than those in 2019, in both types of cultivation. Consequently, we could assume that any increase or decrease in the yields in intercropping cultivation was not a primary factor contributing to the increase in the TKWs of the spring oat Dunav and the winter triticale Odisej, when they had been intercropped with pea. We also observed that the TKWs and the changes in yield were not associated in the spring wheat Nataša or the winter oat Jadar. The high temperatures at the time of flowering in 2018 had decreased the yields of the spring wheat Nataša and the winter oat Jadar, in both types of cultivation, but their TKWs did not differ during the two growing seasons and did not exhibit any advantage in intercropping cultivation. The possibility of no correlations existing between the yield and the TKW under unfavorable growing conditions had also been reported in previous studies, suggesting that other yield components, such as the number of grains, could compensate for the yield reduction, instead of the TKW [43,44]. The GLM conducted in our study showed that the mechanisms affecting the TKW were variety specific and dependent on the interaction between variety and cultivation practices.

We also noted that extreme fluctuations in climatic factors during the flowering and grain-filling periods decreased the yield variations in the winter-rye, intercropped Savo (<0.23 t/ha), as compared to the results in pure stand cultivation (>1.16 t/ha). This indicated that for some cultivars, lowering the sowing density and the interaction with legume intercrops could result in higher yield stability under fluctuating climatic conditions. Consequently, more investigations are needed to explain the crosstalk of the signaling pathways that affect the relationships between given parameters.

Finally, previous studies had showed that a reduction in the N/carbohydrate ratio, which was affected by starch, was positively correlated with TKW [45]. Carbohydrates in the grains are primarily a result of photosynthesis during the grain filling-period and are the main components of endosperm, together with proteins. Consequently, the N/carbohydrate ratio in grains plays an important role in determining the TKW, and the factors affecting the N/carbohydrate ratio should also be addressed in future studies considering the factors affecting yield and TKWs in intercropping cultivation.

### 3.2. Potential of Cereal–Pea Intercropping Systems for Regulating Powdery Mildew and Leaf Rust Infection

Intercropping cultivation has been reported as a promising tool for pest control management, but unanswered questions remain regarding the relationships among the level of disease infection, final yield results, and quality. The beneficiary effects of intercropping cultivation on crop protection cannot be fully understood without understanding how different levels of pathogenic infection could impact yield losses in a single variety. The yield responses to a wide range of infections in susceptible varieties have rarely been addressed in the studies, and we indicated that the relationship between the level of pathogenic infection and yield performance was not straightforward and was highly dependent on the yield potential of the variety. In our study, the winter wheat Ilina, infected with powdery mildew (DI = 35%) in 2018, expressed an almost optimal yield potential (7.6 t/ha), alongside the spring wheat Nataša (6.4 t/ha) which had not been infected by obligate pathogens. The yield results of these two varieties were the same under low disease infection and intercropping conditions in 2018, indicating that the pressure of diseases on yield should not be analyzed individually without considering the overall yield potential and the genotype stability, under diverse environmental conditions.

Previous studies have indicated that obligate pathogens restricted the normal remobilization from assimilated to developing grains and decreased the N remobilization efficacy. However, the effects of foliar diseases on the photosynthesis of wheat leaves with different N contents is extremely limited. Foliar N content has been reported as a major determinant of photosynthesis rate. The light-saturated photosynthesis (Pmax) of healthy leaves has also been shown to be significantly higher in comparative studies of high versus low N treatments [28,46]. However, Carretero et al. [44] reported that changes in the leaf nitrogen concentration did not modify the effects of leaf rust on the net-photosynthesis since leaf rust could only affect the net-photosynthesis through non-stomatal events, such as chlorophyll reduction. Carretero et al. [44] also suspected that leaf rust may affect light interception, rather than radiation-usage efficiency, at the crop level. The independence of the metabolic actions of leaf rust and foliar nitrogen content on net-photosynthesis could be one of the reasons why there were no differences in the yield results among the varieties having different levels of obligate pathogenic infection in pure stand cultivation. However, we should also note that the effects of the pathogens and N availability on the net-photosynthesis should not be analyzed individually without considering the overall yield potential and the genotype stability under different environmental conditions. The higher temperatures at the time of flowering in 2018 decreased the yields in the spring wheat Nataša in both types of cultivation, while the winter wheat Ilina had a higher yield in 2018 than in 2019, regardless of the type of cultivation.

Changes in the predominance of the obligate pathogens on individual susceptible varieties are also an obstacle in pathogen prediction and management. The predominance of leaf rust over powdery mildew, and vice versa, on the winter triticale Odisej was not consistent across the two growing seasons in pure stand cultivation. This supported the results of Jevtić et al. [29], which indicated that the reaction of the genotypes to climatic factors in certain phenological stages could have had a strong impact on the interactions between obligate pathogens and their predominance in a single susceptible variety. Consequently, the effects of combined abiotic and biotic stressors on plant responses to pathogenic infection should be comprehensively addressed in the future.

Previous studies have shown that increased metabolite pools within the host cells of wheat plants could stimulate higher susceptibility to *P. triticina* [47,48,49]. Nitrogen could influence pathogenic infection either by increasing the N compounds necessary for pathogenic growth [48] or by enhancing the aboveground biomass that could create a positive crop microclimate for fungal diseases [50,51]. The enhancement of the N usage in the intercropping systems in our study did not result in increased leaf rust infection; instead, the opposite was found. Consequently, our study supported previous reports that intercropping could be considered a promising tool in pathogenic control through the allelopathic interactions and the physiognomies of the intercrops [52]. However, since N could also enhance the plant defense response, as shown by Solomon et al. [53] and Tavernier et al. [54], a better understanding of how N usage affects nitrogen dynamics, yield results, the end-use quality of cereal crops, and pathogenic control is needed in order to explain the benefits of intercropping over pure stand cultivation.

### 3.3. Effect of Intercropping Systems on Nitrogen Usage of Cereal Crops and Contribution of Cereal Crops to the Land Equivalent Ratio

In our study, the LERs were higher than 1 in all the intercropping systems, but the contributions of the different cereal crops to the LERs were significantly varied within each year and between years. This indicated not only the specificities in the competitive, complementary, and facilitative interactions between the intercropped varieties, but also the complexity of their reactions to the combined abiotic and biotic stressors of the environment. Brooker et al. [7] indicated that there was high uncertainty in the production of a single standardized product in intercropping systems, leading to a limitation of their usage on a large scale. The improved productivity of intercropping systems was still referenced only in terms of yield-per-unit area and was associated with the complementary use of resources by intercrops for the facilitation and/or increase in pest regulation [7,55].

In our study, the enhancement of N usage in intercropping cultivation resulted in higher crude protein contents and supported previous studies showing the beneficial effects of cereal–legume intercropping on the protein accumulation due to elevated N usage. In our study, the crude protein contents were higher in almost all cereal crops during both growing seasons in intercropping cultivation. Previous studies had indicated that the advantageous mechanisms of intercropping cultivation for N usage efficacy were complex and resulted from competitive, complementary, and facilitative interactions between grain legumes and cereal intercrops [3]. However, there remain unanswered questions regarding the mechanisms that provide an advantage for crude protein contents in intercropping systems. 

Jensen [56] indicated that the intercropping advantage in the pea–barley intercrop was primarily related to the complementary use of soil with inorganic and atmospheric N sources, rather than a facilitative effect, in which symbiotically fixed N_2_ was made available to the barley. There have also been reports indicating that intercropped legumes were capable of partially transferring fixed symbiotic N to intercropped cereals, but the amount of N transfer varied widely in the studies [57,58]. Jensen [59] reported that the N nutrition of an intercropped non-legume could be associated with N that had been deposited in the pea rhizosphere during growth. However, we should note that cereal crops, as stronger competitors for soil-based N, also acquired a much larger proportion of the soil-based N, as compared to their abundance in the intercrops [3]. Our study supported the report of Jensen et al. [3], but our results also indicated that cereal crops would not necessarily experience an increase in crude protein content, as found in the spring oat Dunav in 2019.

## 4. Materials and Methods

A field trial was conducted at the experimental field of the Institute of Field and Vegetable Crops in Novi Sad, Serbia, over two growing seasons, 2017/2018 and 2018/2019. Winter wheat Ilina, spring wheat Nataša, winter oat Jadar, spring oat Dunav, winter triticale Odisej, and winter rye Savo were used in the study. All cereal crops sown in the autumn were intercropped with winter pea Kosmaj, while spring pea NS Junior was used as intercrop for spring sown cereals. All varieties were released by the Institute of Field and Vegetable Crops in Novi Sad, Serbia. The optimal time for sowing winter varieties was October and for spring varieties, March in both 2018 and 2019.

The soil type was a slightly carbonated loamy chernozem. The sowing preparation included ploughing, disc-harrowing, and cultivating. Fertilization was conducted in October with MAP 12:52:0 (200 kg/ha), before both winter and spring sowing. Mixed intercropping systems were used. The cereal crops and the peas were sown in two different passes. First, the peas were sown at the desired depth, and afterwards, the cereal crops were sown at a shallower depth. Sowing depth for pea (winter and spring) was 4–5 cm while for cereal crops, it was 3–4 cm. A field trial was arranged in accordance with a randomized block design, with four replications. The plot size of each replicate was 5 m^2^. The cereals were planted at 30% of the conventional sowing rate. The sowing rates of cereal crops in conventional production are Ilina: 220 kg/ha; Nataša: 240 kg/ha; Jadar: 158 kg/ha; Dunav: 196 kg/ha; Odisej: 275 kg/ha; and Savo: 153 kg/ha. Sowing rate of peas in intercropping was 70% of conventional rate. Sowing rate in conventional production of both Kosmaj and NS Junior was 140 kg/ha.

### 4.1. Disease Assessments

The disease indices (DI%) of leaf rust and powdery mildew were scored at the 71–73 BBCH (kernel watery; early milk) growth stage, which were known to be highly related to yield [60]. DIs were calculated by taking into consideration disease incidence and average disease severity [61] and using the Townsend–Heuberger formula (Equation (1)). Disease severity was defined as the percentage of relevant host tissues or organs, covered by symptoms [61]. A total of 10 plants were sampled from each plot (standalone) and per each crop (intercropping) (40 plants per crop). The upper three leaves (the flag leaf “F”, and the two leaves below) were scored for the presence of powdery mildew and rusts for each plant. Assessments of leaf disease severity were made using a modified Cobb’s scale [62] (Table 3).

(1) $$\mathrm{DI}\;\left(\%\right)=\left(\sum^{\;}\left(n\times v\right)/i\times N\right)\times 100$$

*v*—class of infection;

*i*—highest class of infection (9 in this case);

*n*—number of plants in each class;

*N*—total amount of plants.

### 4.2. Yield and Crude Protein

The harvest occurred at the beginning of July for both winter- and spring-planted crops. All crops had similar maturity time periods, but cereal crops needed a high rotational speed of the threshing drum, causing the beans to break up, so peas were manually removed from the experimental plots before the harvest of cereal crops. Harvest of the cereal crops was performed using a combine harvester, and yield was measured per each plot at 15% water content. The pea plants that were pulled up by hand and collected in sacks in the field were also harvested using a combine harvester. The plants collected in sacks were delivered to the reel of the combine harvester, and the seed was gathered for yield measurement at 15% water content.

The crude protein contents of cereal crops (wheat, triticale, ray, and oat) were determined according to the improved Kjeldahl method using a Kejltec 2300 (Foss, Hillerød, Denmark). A total of 10 spikes of each plot were air-dried, the grains were thoroughly mixed, and samples of 50 g were taken for protein analysis. Crop samples were ground in the AB-30 laboratory mill (Falling Number, Stockholm, Sweden), and thereafter, approximately 0.7 g of each cereal/crop sample was measured in duplicate in a digestion flask and digested in sulfuric acid, ammonia was distilled, and excess acid was titrated (AACC 2000 method 46-10). The conversion factor that was used for all samples was 6.25, except for wheat at 5.7. 

### 4.3. Land Equivalent Ratio (LER)

LER based on yield was defined as relative land-area-required as sole crops to produce the same yields as intercropping [63]. An LER greater than 1 indicated good land usage; for example, a total of 1.4 ha of sole cropping area would be required to produce the same yields as 1 ha of the intercropped system when the LER equaled 1.4. The land equivalent ratio based on yield was the sum of the partial LER values for cereals and legumes (Equation (2)).

(2) $${\mathrm{LER}}_{\mathrm{yield}}=\frac{{\mathrm{Yield}}_{\mathrm{Cereal-Intercrop}}}{{\mathrm{Yield}}_{\mathrm{Cereal-Standalone}}}+\frac{{\mathrm{Yield}}_{\mathrm{Legume-Intercrop}}}{{\mathrm{Yield}}_{\mathrm{Legume-Standalone}}}$$

### 4.4. Climatic Conditions

**Figure 7:**
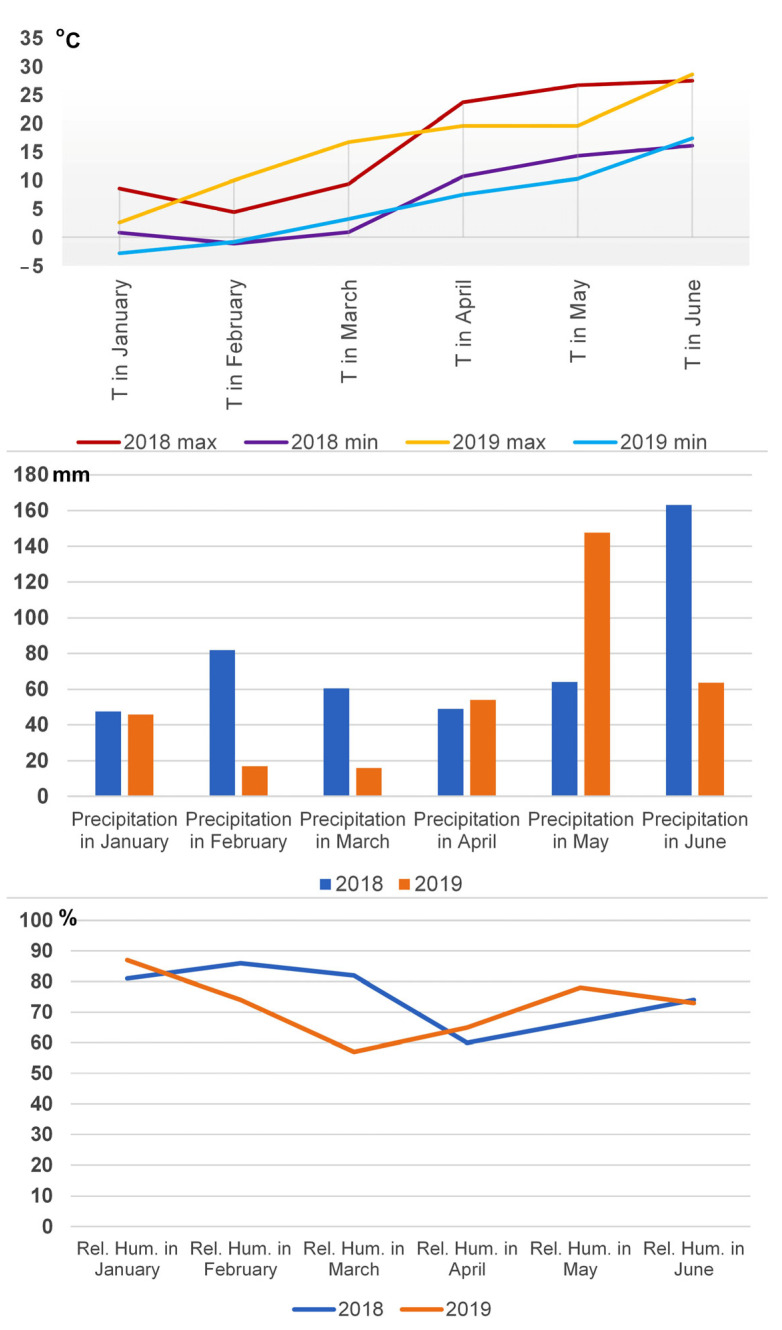
Climatic conditions in 2018 and 2019 at the Rimski Šančevi locality.

Environmental conditions were monitored for the experimental site. The data originated from the Republic Hydrometeorological Service of Serbia (http://www.hidmet.gov.rs/, accessed on 20 February 2023). The climatic factors in our study showed extreme fluctuations during the time of flowering and grain-filling period that were known to be highly associated with yield and TKW results (Figure 7). In 2018, the average temperature in May (20.4 °C) exceeded the 15-year average of 17.4 °C, while in 2019 (14.7 °C), it was lower than the 15-year average. Two growing seasons were also characterized by the extreme fluctuation of total rainfall at the time of flowering and grain-filling period. In 2018, total rainfall (64.2 mm) was below the 15-year average (93.73 mm), while in 2019 (147.6 mm), it was the opposite.

### 4.5. Statistical Methods

The effects of year, variety, cultivation practice, DI of leaf rust, and DI of powdery mildew on yield, TKW, and crude protein were examined using a general linear model (GLM) as an ANOVA procedure. Disease indices of leaf rust and powdery mildew were used as continuous predictors, while variety, year, and cultivation practices were used as categorical predictors. Since abiotic and biotic factors could be correlated (multicollinearity), the stepwise effect selection in GLM was also applied. The alpha level to enter and alpha level to remove the influencing factors were set by default to 0.15 in the stepwise variable selection, since it was reported that an alpha level of 0.05 could fail to identify important variables [64]. Tukey’s pairwise comparisons with 95% confidence were used to provide information on which means were significantly different.

## 5. Conclusions

Understanding the factors affecting the variable relationships among yield, yield components, and crude protein in different cultivation systems could inform not only how the change in one variable impacts a change in others, but also how competitive, complementary, and facilitative interactions between grain legumes and cereal intercrops affect the overall performance of cereal crops in changing agro-ecological conditions. Although our study supported the previously published trends concerning the yield and quality parameters of cereal crops cultivated in intercropping systems, it also indicated that changes in yield, TKW, and crude protein, as well as their relationships, were cultivar specific and were not the same for all cereal crops cultivated under same agro-ecological conditions.

In addition, our study indicated that more investigations should be focused on the thresholds of infection, above which a significant contribution could be expected of intercropping systems on disease management. Consequently, the main conclusions of our study were the following:

1. The effect of intercropping on yield and TKW was not straightforward. The changes in TKW in a single variety cultivated using different practices were not dependent on the year to the same extent as the yield.
2. Intercropping could decrease the yield variation under extreme fluctuations of climatic factors during the flowering and grain-filling periods.
3. The relationships between the level of pathogenic infection and yield results were not straightforward and were highly dependent on the yield potentials of the cultivars.
4. The contributions of the cereal crops to the LER differed within and across the years.
5. Year, variety, cultivation practices, and their interactions (year × variety × cultivation) were all determined as significantly influencing factors (*p* < 0.001) on the crude protein of cereal crops.

## Figures and Tables

**Table 1:** The most influential factors on yield, TKW, crude protein, and disease indices of powdery mildew and leaf rust in wheat, rye, oat, and triticale, all cultivated in intercropping and standalone cultivation systems.

	Yield t/ha	TKW g	Crude Protein%	Powdery Mildew%	Leaf Rust %
Powdery mildew	0.063	-	-	-	-
Leaf rust	0.147	-	-	-	-
Year	0.003	0.006	<0.001	0.202	0.009
Variety	<0.001	<0.001	<0.001	<0.001	<0.001
Cultivation	<0.001	<0.001	<0.001	<0.001	<0.001
Seeding time	-	-	-	-	-
Year × Variety	<0.001	<0.001	<0.001	<0.001	0.004
Year × Cultivation	0.192	-	<0.001	0.669	0.503
Variety × Cultivation	0.428	<0.001	<0.001	0.029	<0.001
Year × Variety × Cultivation	0.033	-	<0.001	0.021	0.001

**Table 2:** Averages of yield, TKW, crude protein, disease index of leaf rust, and disease index of powdery mildew in individual varieties of wheat, rye, oat, and triticale cultivated in 2018 and 2019 in intercropping and standalone cultivation systems.

Variety ^1^	Cultivation Practice	Yield t/ha	SE of Mean	Yield t/ha	SE of Mean	TKW g	SE of Mean	TKW g	SE of Mean	Crude Protein%	SE of Mean	Crude Protein %	SE of Mean	PM ^2^ %	SE of Mean	PM ^2^ %	SE of Mean	LR ^3^ %	SE of Mean	LR ^3^ %	SE of Mean
		2018		2019		2018		2019		2018		2019		2018		2019		2018		2019	
Dunav	Intercropping	3.9	0.37	6.5	0.16	34.2	0.46	34.5	1.08	10.4	0.36	6.1	0.14	7.5	1.44	8.7	1.25	0.0	0	0.0	0
	Standalone	5.9	0.28	9.5	0.04	29.9	0.49	30.5	0.29	8.9	0.21	6.7	0.17	5.0	2.04	12.5	2.50	0.0	0	0.0	0
Ilina	Intercropping	4.7	0.20	4.2	0.19	41.8	0.72	35.4	0.42	8.9	0.12	9.1	0.12	10.0	0	12.5	2.50	0.0	0	0.0	0
	Standalone	7.6	0.08	7.4	0.37	39.4	1.55	35.5	0.45	7.4	0.09	6.9	0.09	35.0	2.89	30.0	4.08	10.0	4.08	2.5	2.50
Jadar	Intercropping	3.6	0.18	4.3	0.36	28.7	0.96	28.8	0.73	8.7	0.16	7.6	0.19	3.7	2.39	5.0	2.04	0.0	0	0.0	0
	Standalone	6.3	0.52	7.9	0.30	28.7	1.10	28.5	0.55	7.9	0.12	6.2	0.07	15.0	2.89	6.2	2.39	0.0	0	0.0	0
Nataša	Intercropping	4.2	0.19	4.4	0.29	38.4	0.51	39.2	0.34	10.4	0.04	8.2	0.07	0.0	0	0.0	0	0.0	0	5.0	2.89
	Standalone	6.4	0.23	7.5	0.10	40.3	1.43	38.9	0.69	9.1	0.04	7.3	0.06	0.0	0	3.7	2.39	0.0	0	12.5	4.79
Odisej	Intercropping	4.6	0.09	3.2	0.21	48.5	0.89	49.6	0.47	12.1	0.06	9.6	0.13	0.0	0	6.2	1.25	8.7	1.25	6.2	4.73
	Standalone	7.8	0.15	6.4	0.46	45.8	0.55	46.6	0.39	9.5	0.04	7.4	0.09	10.0	4.08	15	5.00	15.0	2.89	5	5.00
Savo	Intercropping	5.4	0.21	5.2	0.21	32.1	0.97	29.5	0.76	9.6	0.07	6.8	0.14	0.0	0	0.0	0	8.7	1.25	11.2	3.15
	Standalone	8.3	0.39	6.9	0.38	28.4	0.83	28.1	0.62	7.2	0.07	5.6	0.09	0.0	0	16.2	2.39	12.5	2.50	27.5	2.50
	Mean Intercropping	4.4		4.6		37.3		36.2		10.0		7.9		3.5		5.4		2.9		3.7	
	Mean Standalone	7.1		7.6		35.4		34.7		8.3		6.7		10.8		13.9		6.2		7.9	

^1^ Dunav is spring oat; Ilina is winter wheat; Jadar is winter oat; Nataša is spring wheat; Odisej is winter triticale; and Savo is winter rye. ^2^ Disease index of powdery mildew; ^3^ Disease index of leaf rust.

**Table 3:** Assessments of leaf disease severity.

**The Score**	0	1	2	3	4	5	6	7	8	9
**Level of Infection**	no infection	1–10%	11–20%	21–30%	31–40%	41–50%	51–60%	61–70%	71–80%	≥81%

## Data Availability

Data are reported within the article and Supplementary Material.

## Supplementary Materials

The following supporting information can be downloaded at: https://www.mdpi.com/article/10.3390/plants12112067/s1, Figure S1. Box plots showing yield and protein contents of the winter and spring pea varieties, Kosmaj and Junior, respectively, in intercropping and standalone cultivation systems in 2018 and 2019; Table S1: The most influential factors on yield and crude protein of two pea cultivars with intercropping and standalone cultivation practices; Table S2: Pairwise mean differences in yield, TKW, crude protein, disease indices of powdery mildew and leaf rust for variety × cultivation interactions obtained by using Tukey’s method for multiple comparisons; Table S3: Pairwise mean differences in disease indices of leaf rust and powdery mildew for year × variety × cultivation interactions obtained by using Tukey’s method for multiple comparisons; Table S4: Pairwise mean differences in yield for year × variety × cultivation interactions obtained by using Tukey’s method for multiple comparisons; Table S5: Pairwise mean differences in crude protein for year × variety × cultivation interactions obtained by using Tukey’s method for multiple comparisons; Table S6: Land equivalent ratio of yield of wheat, rye, oat, triticale, and pea in 2018 and 2019.
