# Supplementary material for: Cereal–Pea Intercropping Reveals Variability in the Relationships among Yield, Quality Parameters, and Obligate Pathogens Infection in Wheat, Rye, Oat, and Triticale, in a Temperate Environment
Source: Plants (Basel). 2023 May 23;12(11):2067. doi: 10.3390/plants12112067 (PMC10255238; doi:10.3390/plants12112067)
Supplement: Supplementary file 1 [file plants-12-02067-s001.zip › plants-2294204-supplementary.pdf]

Supplementary Information for:

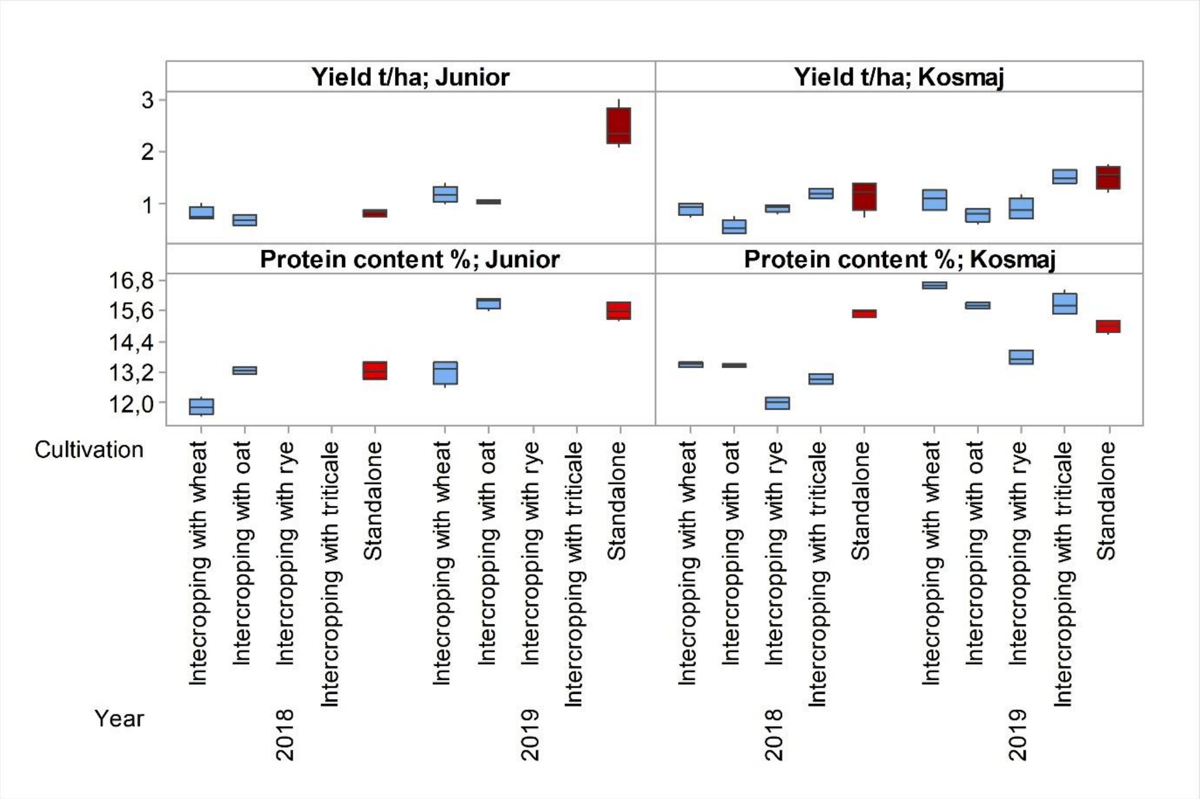

Figure S1. Box plots showing yield and protein contents of the winter and spring pea varieties, Kosmaj and Junior, respectively, in intercropping and standalone cultivation systems in 2018 and 2019.

Table S1. The most influencing factors on yield and crude protein of two pea cultivars cultivated with intercropping and standalone cultivation practices

Variety	Cultivation	Yield (t ha^{-1})		Protien content %	
		2018	2019	2018	2019
Kosmaj	Intercropping with wheat	0,88	1,05	13,47	16,59
	Intercropping with oat	0,52	0,76	13,43	15,77
	Intercropping with rye	0,89	0,88	11,94	13,74
	Intercropping with triticale	1,17	1,48	12,89	15,84
	Standalone	**1,14**	**1,48**	**15,51**	**14,98**
NS Junior	Intercropping with wheat	0,79	1,16	11,81	13,19
	Intercropping with oat	0,66	1,02	13,22	15,92
	Standalone	**0,79**	**2,42**	**13,21**	**15,62**
GLM		P		P	
	Year	<0,001		<0,001	
	Variety	0,012			
	Cultivation	<0,001			
	Seeding time				
	Year × Variety	<0,001			
	Year × Cultivation	<0,001			
	Variety × Cultivation			<0,001	
	Year × Variety × Cultivation			<0,001	

Table S2. Pairwise mean differences in yield, TKW, crude protein, disease indices of powdery mildew and leaf rust for variety × cultivation interactions obtained by using Tukey's method for multiple comparisons

**Response: Yield**			
**Variety × Cultivation**	**N**	**Fitted Mean**	**Grouping**
Savo Standalone	8	7.92079	A
Dunav Standalone	8	7.61284	A
Nataša Standalone	8	7.16070	A
Odisej Standalone	8	7.08696	A
Jadar Standalone	8	6.94373	A
Ilina Standalone	8	6.88497	A B
Savo Intercropping	8	5.58715	B C
Dunav Intercropping	8	5.10546	C D
Nataša Intercropping	8	4.48773	D E
Ilina Intercropping	8	4.32435	D E
Odisej Intercropping	8	4.05949	E
Jadar Intercropping	8	3.93945	E
**Reponse: TKW**			
**Variety × Cultivation**	**N**	**Fitted Mean**	**Grouping**
**Odisej Intercropping**	**8**	**49.0625**	**A**
**Odisej Standalone**	**8**	**46.2125**	**B**
Nataša Standalone	8	39.5875	C

Nataša Intercropping	8	38.8500	C
Ilina Intercropping	8	38.6500	C
Ilina Standalone	8	37.4875	C
**Dunav Intercropping**	**8**	**34.3625**	**D**
Savo Intercropping	8	30.8500	E
**Dunav Standalone**	**8**	**30.2375**	**E**
Jadar Intercropping	8	28.7750	E
Jadar Standalone	8	28.6375	E
Savo Standalone	8	28.2500	E
**Response: Crude protein**			
**Variety × Cultivation**	**N**	**Fitted Mean**	**Grouping**
**Odisej Intercropping**	**8**	**10.8393**	**A**
Nataša Intercropping	8	9.2862	B
Ilina Intercropping	8	9.0601	B
**Odisej Standalone**	**8**	**8.4546**	**C**
Dunav Intercropping	8	8.2334	C D
**Savo Intercropping**	**8**	**8.2213**	**C D**
Nataša Standalone	8	8.2186	C D
Jadar Intercropping	8	8.1457	C D
Dunav Standalone	8	7.7871	D
Ilina Standalone	8	7.1942	E
Jadar Standalone	8	7.1013	E
**Savo Standalone**	**8**	**6.3918**	**F**
**Response: DI Powdery mildew**			
**Variety × Cultivation**	**N**	**Fitted Mean**	**Grouping**
Ilina Standalone	8	32.500	A
Odisej Standalone	8	12.500	B
Ilina Intercropping	8	11.250	B C
Jadar Standalone	8	10.625	B C D
Dunav Standalone	8	8.750	B C D E
Dunav Intercropping	8	8.125	B C D E
Savo Standalone	8	8.125	B C D E
Jadar Intercropping	8	4.375	C D E F
Odisej Intercropping	8	3.125	D E F
Nataša Standalone	8	1.875	E F
Savo Intercropping	8	0.000	F
Nataša Intercropping 8	8	-0.000	F
**Response: DI Leaf rust**			
**Variety × Cultivation**	**N**	**Fitted Mean**	**Grouping**
Savo Standalone	8	20.00	A
Savo Intercropping	8	10.00	B
Odisej Standalone	8	10.00	B
Odisej Intercropping	8	7.50	B C
Nataša Standalone	8	6.25	B C
Ilina Standalone	8	6.25	B C
Nataša Intercropping	8	2.50	B C
Dunav Standalone	8	-0.00	C
Jadar Standalone	8	-0.00	C
Ilina Intercropping	8	-0.00	C
Jadar Intercropping	8	-0.00	C

Dunav Intercropping	8	-0.00	C

Means that do not share a letter are significantly different.

Table S3. Pairwise differences of disease indices of leaf rust and powdery mildew means for year × variety × cultivation interaction obtained by using Tukey's method for multiple comparisons

**Response: DI Leaf rust**			
**Year × Variety × Cultivation**	**N**	**Fitted Mean**	**Grouping**
**2019 Savo Standalone**	**4**	**27.50**	**A**
2018 Odisej Standalone	4	15.00	A B
2018 Savo Standalone	4	12.50	B C
2019 Nataša Standalone	4	12.50	B C
**2019 Savo Intercropping**	**4**	**11.25**	**B C**
2018 Ilina Standalone	4	10.00	B C
2018 Odisej Intercropping	4	8.75	B C
2018 Savo Intercropping	4	8.75	B C
2019 Odisej Intercropping	4	6.25	B C
2019 Odisej Standalone	4	5.00	B C
2019 Nataša Intercropping	4	5.00	B C
2019 Ilina Standalone	4	2.50	B C
2018 Nataša Standalone	4	0.00	C
2018 Jadar Standalone	4	0.00	C
2018 Dunav Intercropping	4	0.00	C
2018 Dunav Standalone	4	0.00	C
2018 Nataša Intercropping	4	0.00	C
2018 Ilina Intercropping	4	0.00	C
2019 Dunav Standalone	4	- 0.00	C
2019 Dunav Intercropping	4	- 0.00	C
2019 Jadar Intercropping	4	- 0.00	C
2019 Jadar Standalone	4	- 0.00	C
2018 Jadar Intercropping	4	- 0.00	C
2019 Ilina Intercropping	4	- 0.00	C
**Reponse:DI Powdery mildew**			
**Year × Variety × Cultivation**	**N**	**Fitted Mean**	**Grouping**
**2018 Ilina Standalone**	**4**	**35.00**	**A**
**2019 Ilina Standalone**	**4**	**30.00**	**A**
2019 Savo Standalone	4	16.25	B
2018 Jadar Standalone	4	15.00	B C
2019 Odisej Standalone	4	15.00	B C
**2019 Ilina Intercropping**	**4**	**12.50**	**B C**
2019 Dunav Standalone	4	12.50	B C
**2018 Ilina Intercropping**	**4**	**10.00**	**B C D**
2018 Odisej Standalone	4	10.00	B C D
2019 Dunav Intercropping	4	8.75	B C D
2018 Dunav Intercropping	4	7.50	B C D
2019 Odisej Intercropping	4	6.25	B C D
2019 Jadar Standalone	4	6.25	B C D
2018 Dunav Standalone	4	5.00	B C D
2019 Jadar Intercropping	4	5.00	B C D
2019 Nataša Standalone	4	3.75	C D

2018 Jadar Intercropping	4	3.75	C D
2019 Savo Intercropping	4	0.00	D
2018 Savo Intercropping	4	0.00	D
2019 Nataša Intercropping	4	0.00	D
2018 Savo Standalone	4	0.00	D
2018 Nataša Intercropping	4	-0.00	D
2018 Nataša Standalone	4	-0.00	D
2018 Odisej Intercropping	4	-0.00	D

Table S4. Pairwise differences of yield means for year × variety × cultivation interaction obtained by using Tukey's method for multiple comparisons

**Response: Yield**			
**Year × Variety × Cultivation**	**N**	**Fitted Mean**	**Grouping**
**2019 Dunav Standalone**	4	9.33024	A
2018 Savo Standalone	4	8.64496	A B
2018 Odisej Standalone	4	7.98206	A B C
**2019 Jadar Standalone**	4	7.86121	A B C
**2019 Nataša Standalone**	4	7.81548	A B C
2019 Savo Standalone	4	7.19662	B C D
2018 Ilina Standalone	**4**	7.03206	B C D E F
2019 Ilina Standalone	4	6.73787	B C D E F
**2018 Nataša Standalone**	**4**	6.50593	C D
2019 Dunav Intercropping	4	6.40847	C D E
2019 Odisej Standalone	4	6.19186	D E F
**2018 Jadar Standalone**	4	6.02625	D E F G
**2018 Dunav Standalone**	4	5.89545	D E F G
2018 Savo Intercropping	4	5.66600	D E F G H
2019 Savo Intercropping	4	5.50831	D E F G H
2018 Odisej Intercropping	4	4.86400	E F G H I
2019 Nataša Intercropping	4	4.65379	F G H I J
2018 Ilina Intercropping	4	4.60447	G H I J
2018 Nataša Intercropping	4	4.32168	H I J
2019 Jadar Intercropping	4	4.27920	H I J
2019 Ilina Intercropping	4	4.04424	H I J
2018 Dunav Intercropping	4	3.80246	I J
2018 Jadar Intercropping	4	3.59969	I J
2019 Odisej Intercropping	4	3.25497	J

Means that do not share a letter are significantly different.

Table S5. Pairwise differences of crude protein means for year × variety × cultivation interaction obtained by using Tukey's method for multiple comparisons

**Response: Crude protein**			
**Year × Variety × Cultivation**	**N**	**Fitted Mean**	**Grouping**
2018 Odisej Intercropping	4	12.1045	A
2018 Dunav Intercropping	4	10.3976	B
2018 Nataša Intercropping	4	10.3781	B
2018 Savo Intercropping	4	9.6175	C

2019 Odisej Intercropping	4	9.5741	C D
2018 Odisej Standalone	4	9.5045	C D
2019 Ilina Intercropping	4	9.1345	C D E
2018 Nataša Standalone	4	9.1055	C D E
2018 Ilina Intercropping	4	8.9858	C D E
2018 Dunav Standalone	4	8.8788	D E F
2018 Jadar Intercropping	4	8.7005	E F G
2019 Nataša Intercropping	4	8.1943	F G H
2018 Jadar Standalone	4	7.9916	G H I
2019 Jadar Intercropping	4	7.5909	H I J
2019 Odisej Standalone	4	7.4047	I J K
2018 Ilina Standalone	4	7.3987	I J K
2019 Nataša Standalone	4	7.3316	I J K
2018 Savo Standalone	4	7.2212	J K
2019 Ilina Standalone	4	6.9896	J K
2019 Savo Intercropping	4	6.8252	K L
2019 Dunav Standalone	4	6.6953	K L M
2019 Jadar Standalone	4	6.2109	L M N
2019 Dunav Intercropping	4	6.0692	M N
2019 Savo Standalone	4	5.5625	N

Means that do not share a letter are significantly different.

Table S6. Land equivalent ratio of yield of wheat, rye, oat, triticale and pea in 2018 and 2019.

Year	Seeding time	Variety small grans	Crop	Variety pea	LER small grains	LER pea	LER
2018	Spring	Nataša	Wheat	NS Junior	0,750441	0,95601	1,70646
2018	Spring	Nataša	Wheat	NS Junior	0,6173	1,23454	1,85184
2018	Spring	Nataša	Wheat	NS Junior	0,634687	0,89454	1,52923
2018	Spring	Nataša	Wheat	NS Junior	0,629048	0,88827	1,51731
2018	Spring	Dunav	Oat	NS Junior	0,477251	0,93594	1,41319
2018	Spring	Dunav	Oat	NS Junior	0,663648	0,73771	1,40136
2018	Spring	Dunav	Oat	NS Junior	0,73543	0,68502	1,42045
2018	Spring	Dunav	Oat	NS Junior	0,751344	0,97985	1,7312
2019	Spring	Nataša	Wheat	NS Junior	0,698026	0,47542	1,17345
2019	Spring	Nataša	Wheat	NS Junior	0,558103	0,40668	0,96479
2019	Spring	Nataša	Wheat	NS Junior	0,513847	0,47666	0,99051
2019	Spring	Nataša	Wheat	NS Junior	0,579833	0,56186	1,1417
2019	Spring	Dunav	Oat	NS Junior	0,648569	0,43591	1,08448
2019	Spring	Dunav	Oat	NS Junior	0,680432	0,43673	1,11716
2019	Spring	Dunav	Oat	NS Junior	0,675506	0,39804	1,07354
2019	Spring	Dunav	Oat	NS Junior	0,72875	0,41162	1,14037
2018	Winter	Ilina	Wheat	Kosmaj	0,68145	0,62461	1,30606
2018	Winter	Ilina	Wheat	Kosmaj	0,557823	0,81593	1,37375
2018	Winter	Ilina	Wheat	Kosmaj	0,604526	0,76701	1,37154
2018	Winter	Ilina	Wheat	Kosmaj	0,641156	0,86747	1,50863
2018	Winter	Odisej	Triticale	Kosmaj	0,595237	1,11994	1,71517
2018	Winter	Odisej	Triticale	Kosmaj	0,612349	1,05267	1,66502

2018	Winter	Odisej	Triticale	Kosmaj	0,565481	0,94522	1,5107
2018	Winter	Odisej	Triticale	Kosmaj	0,561267	0,99065	1,55191
2018	Winter	Savo	Rye	Kosmaj	0,595524	0,8203	1,41582
2018	Winter	Savo	Rye	Kosmaj	0,699671	0,69101	1,39068
2018	Winter	Savo	Rye	Kosmaj	0,616305	0,82205	1,43835
2018	Winter	Savo	Rye	Kosmaj	0,684568	0,80545	1,49002
2018	Winter	Jadar	Oat	Kosmaj	0,583485	0,44029	1,02377
2018	Winter	Jadar	Oat	Kosmaj	0,502102	0,41932	0,92142
2018	Winter	Jadar	Oat	Kosmaj	0,546046	0,34943	0,89548
2018	Winter	Jadar	Oat	Kosmaj	0,638693	0,62287	1,26156
2019	Winter	Ilina	Wheat	Kosmaj	0,585627	0,81708	1,4027
2019	Winter	Ilina	Wheat	Kosmaj	0,540955	0,62759	1,16854
2019	Winter	Ilina	Wheat	Kosmaj	0,538375	0,54561	1,08399
2019	Winter	Ilina	Wheat	Kosmaj	0,64605	0,85067	1,49672
2019	Winter	Odisej	Triticale	Kosmaj	0,454595	0,91518	1,36977
2019	Winter	Odisej	Triticale	Kosmaj	0,453497	0,95146	1,40496
2019	Winter	Odisej	Triticale	Kosmaj	0,595401	1,0247	1,6201
2019	Winter	Odisej	Triticale	Kosmaj	0,490856	1,10802	1,59888
2019	Winter	Savo	Rye	Kosmaj	0,739581	0,76063	1,50021
2019	Winter	Savo	Rye	Kosmaj	0,764134	0,48715	1,25129
2019	Winter	Savo	Rye	Kosmaj	0,804049	0,66387	1,46792
2019	Winter	Savo	Rye	Kosmaj	0,658459	0,46028	1,11874
2019	Winter	Jadar	Oat	Kosmaj	0,621446	0,4932	1,11465
2019	Winter	Jadar	Oat	Kosmaj	0,622079	0,55368	1,17575
2019	Winter	Jadar	Oat	Kosmaj	0,460677	0,39846	0,85914
2019	Winter	Jadar	Oat	Kosmaj	0,466622	0,61953	1,08615
